# Long-term complete response in small cell bladder carcinoma treated with carboplatin, etoposide, and atezolizumab

**DOI:** 10.1186/s12894-022-01130-4

**Published:** 2022-11-05

**Authors:** Rei Kamitani, Toshiyuki Ando, Kazuya Hanai, Arihito Tanaka, Fumihiro Kashizaki, Yasutomo Sekido, Ryuichi Mizuno

**Affiliations:** 1 Department of Urology, Isehara Kyodo Hospital, 345, Tanaka, Isehara, Kanagawa 259-1142 Japan; 2 Department of Respiratory Medicine, Isehara Kyodo Hospital, Isehara, Kanagawa Japan; 3 Department of Pathology, Isehara Kyodo Hospital, Isehara, Kanagawa Japan; 4 Department of Urology, Keio University School of Medicine, Tokyo, Japan

**Keywords:** Small cell carcinoma, Bladder cancer, Atezolizumab, Complete response, Case report

## Abstract

**Background:**

Small cell bladder carcinoma (SCBC) is a rare and aggressive malignant tumor with no established treatment guidelines. Its treatment algorithm has been based on the small cell lung cancer (SCLC) guidelines. Metastatic SCBC has poor prognosis (even when treated with platinum-based chemotherapy, which is usually used for extensive-disease SCLC).

**Case presentation:**

Herein, we report a case of a 71-year-old man with SCBC who underwent radical cystectomy and received adjuvant chemotherapy with gemcitabine and cisplatin. However, recurrent tumors were found 6 months postoperatively. The patient was then treated with carboplatin, etoposide, and atezolizumab and achieved complete response. He continues receiving maintenance therapy with atezolizumab monotherapy without any evidence of recurrence over the 12 months follow up.

**Conclusion:**

To our knowledge, this is the first case of metastatic SCBC where carboplatin, etoposide, and atezolizumab achieved long-term complete response.

## Background

Small cell bladder carcinoma (SCBC) is a rare tumor with no established treatment guidelines [1, 2]. Its treatment algorithm has been based on the clinical practice guidelines for small cell lung cancer (SCLC) [3]. Recently, combining chemotherapy with immunotherapy has attracted attention as a treatment option for extensive-disease SCLC [4]. However, few reports have described its efficacy for SCBC [5]. We report the first case of metastatic SCBC where carboplatin, etoposide, and atezolizumab achieved long-term complete response.

## Case presentation

**Fig. 1:**
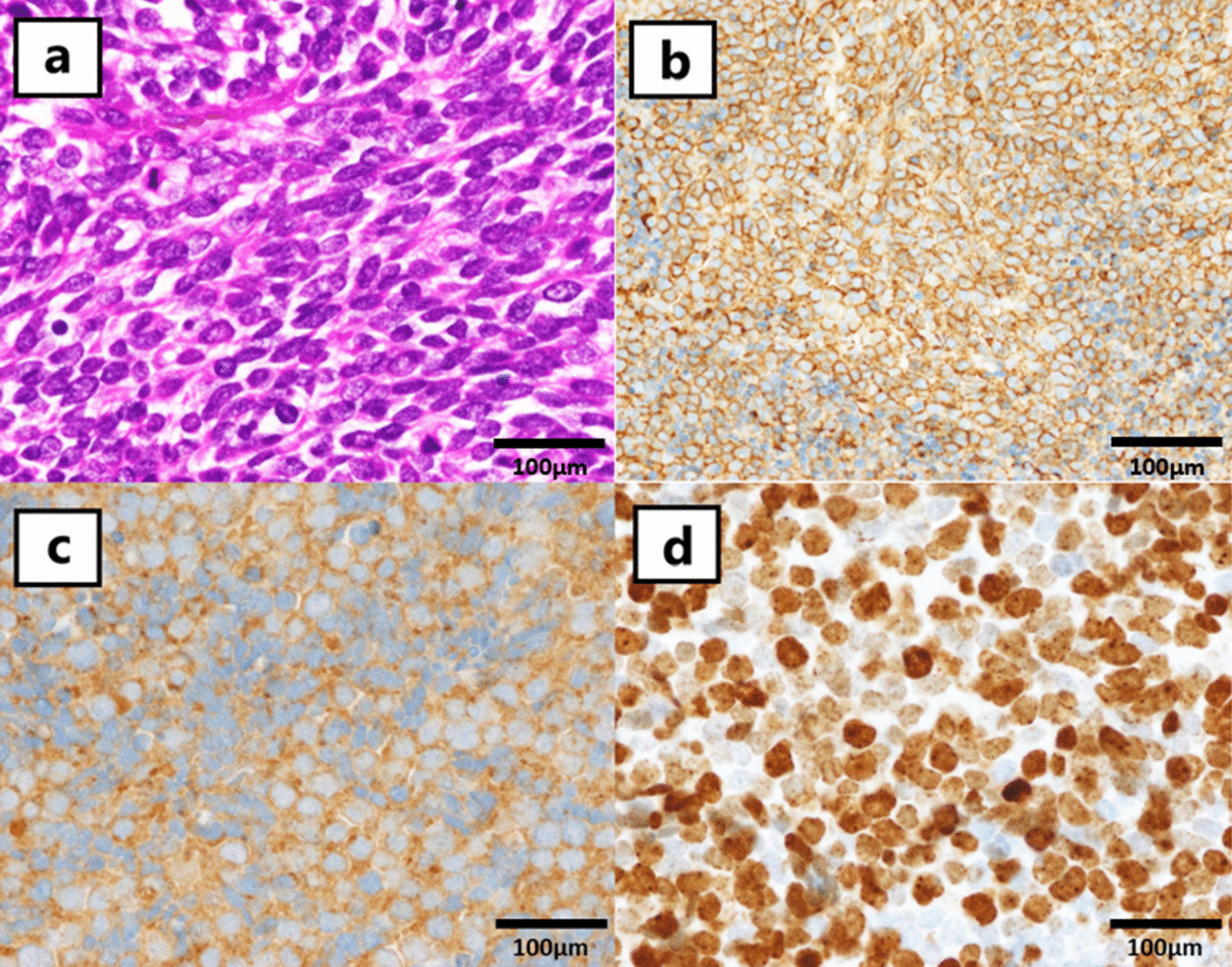
400× magnification of the radical cystectomy specimen. Digital microscopy images were aquired using Olympus BX51 (microscope), Olympus UPlan Apo (objective lens), Olympus DP26 (camera), and Olympus cellSens Standard (imaging software). Hematoxylin and eosin staining exhibited solid growth of spindle-shaped cells (**a**). Immunohistochemically, the cells were positive for CD56 (**b**), synaptophysin (**c**) and ki-67 antibody (> 70%) (**d**)

**Fig. 2:**
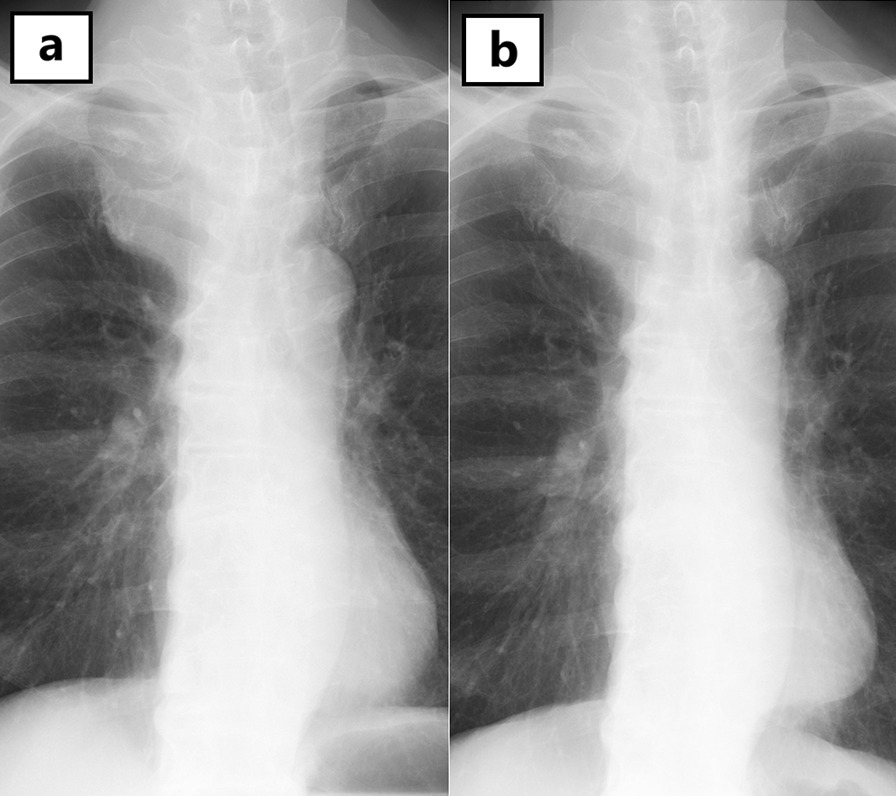
A chest radiograph showed a left tracheal deviation (**a**). A tracheal deviation disappeared on a chest radiograph on the third day of the first cycle (**b**)

**Fig. 3:**
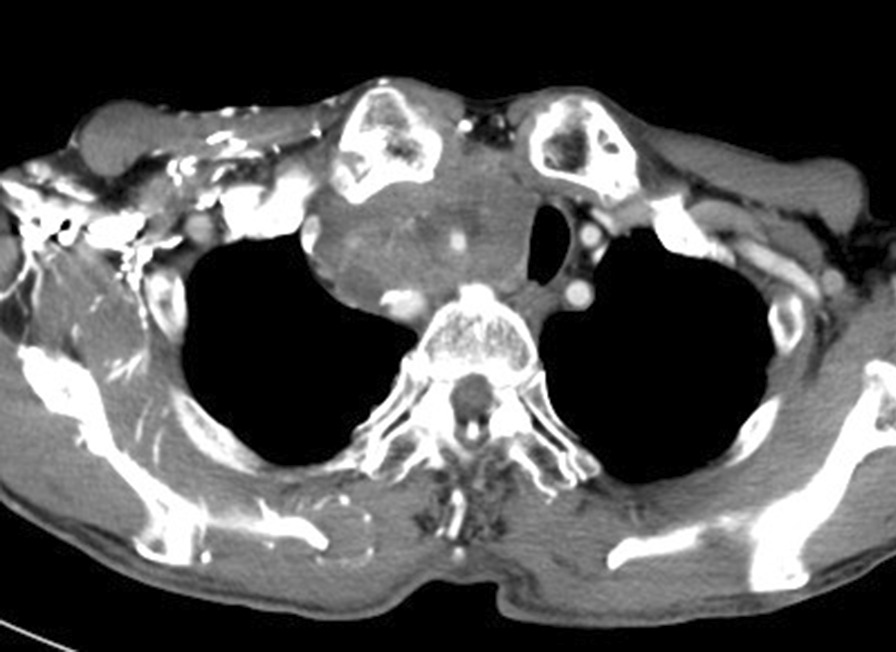
A CT scan showed a 70-mm right-sided infraclavicular lymph node metastasis. A left tracheal deviation was observed due to the tumor

**Fig. 4:**
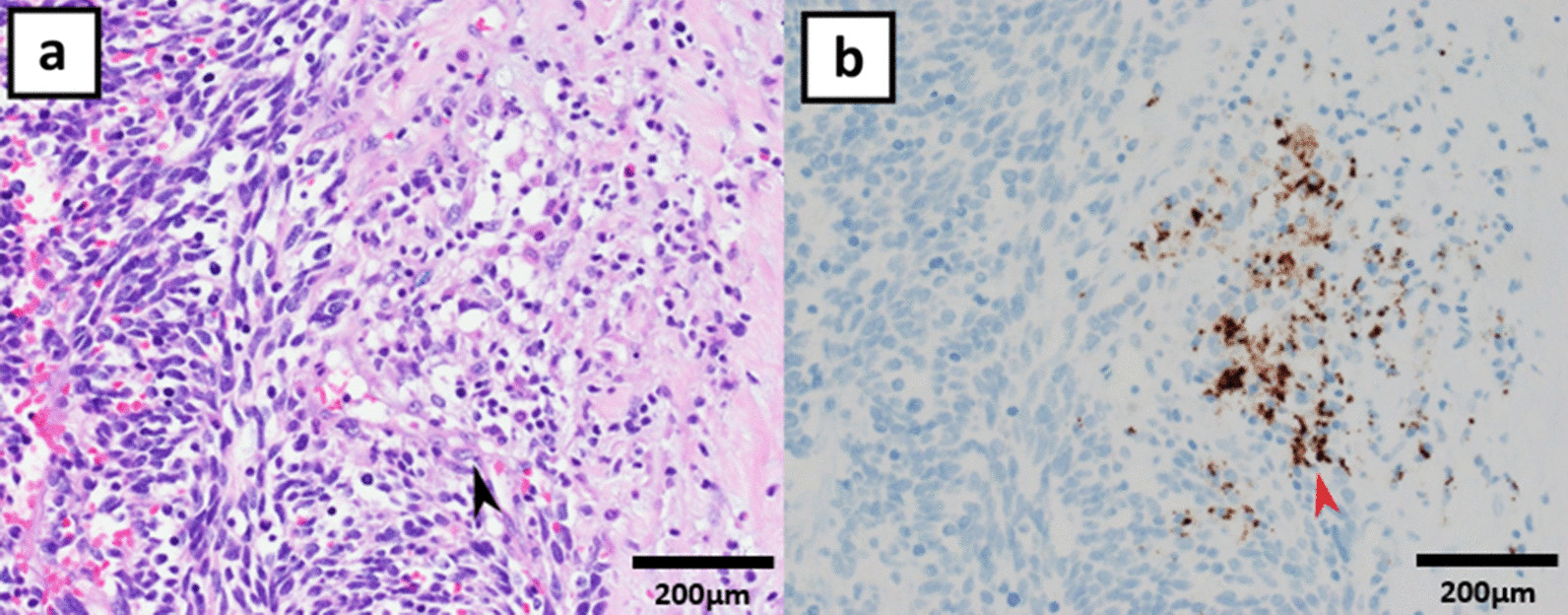
200× magnification of the radical cystectomy specimen. Digital microscopy images were aquired using Olympus BX51 (microscope), Olympus UPlan Apo (objective lens), Olympus DP26 (camera), and Olympus cellSens Standard (imaging software). Hematoxylin and eosin staining exhibited the immune cells that infiltrated the tumor cells (arrow head) (**a**). Immunohistochemically, the immune cells were positive for PD-L1 (red arrow head), whereas the tumor cells were negative for PD-L1 (**b**)

A 71-year-old Japanese man came to our hospital with a chief complaint of hematuria. Diagnosis of muscle invasive bladder cancer without distant metastases was confirmed after imaging tests and transurethral resection of bladder tumor (TURBT). He underwent radical cystectomy and pelvic lymph node dissection; an ileal conduit was also constructed. Microscopically, the tumor exhibited solid growth of spindle-shaped cells with atypia. Immunohistochemically, the cells were positive for CD56, a neural cell adhesion molecule expressed on the cell membrane of neurons, and synaptophysin, a synaptic vesicle glycoprotein that is present in neuroendocrine cells [6]. In contrast, they were negative for CK20, which is a marker of urothelial carcinoma. The cells showed strong intranuclear positivity to ki-67 antibodies (> 70%) (Fig. 1). Invasion beyond the bladder wall and metastasis of one of the thirteen regional lymph nodes that were excised were observed. Diagnosis of SCBC (pT3bN1M0) was confirmed. No component of urothelial carcinoma was found. Additionally, pathological re-examination of TURBT specimen exhibited similar features to the cystectomy specimen, which was pure SCBC. The patient received four cycles of adjuvant chemotherapy with gemcitabine (1000 mg/m^2^, administered intravenously) and cisplatin (70 mg/m^2^, administered intravenously); 6 months after cystectomy, he complained of right arm edema and hoarseness. Left tracheal deviation was observed on a chest radiograph (Fig. 2a); Computed tomography (CT) scan revealed superior vena cava syndrome due to a 70 mm right-sided infraclavicular lymph node metastasis (Fig. 3). Additionally, a 28 mm abdominal wall tumor and a 68 mm pelvic tumor were found. At that time, the serum neuron-specific enolase (NSE) level was 227 ng/ml. The 21-day cycle regimen (including carboplatin, etoposide, and atezolizumab) was administered intravenously, according to the clinical practice guidelines for extensive-disease SCLC [4]. We administered carboplatin (area under the curve, 5 mg min/mL) on the first day, etoposide (100 mg/m^2^) from the first through the third day, and atezolizumab (1200 mg) on the first day. Considering the risk of nausea and loss of appetite, we intravenously administered palonosetron (0.75 mg) on the first day and dexamethasone (6.6 mg) from the first through the third day and orally administered aprepitant (125 mg) once daily on the first day, aprepitant (80 mg) once daily on the second and third day, and metoclopramide (5 mg) three times daily from the third through the seventh day. On the third day of the first cycle, right arm edema and hoarseness had improved. Additionally, tracheal deviation disappeared from the radiograph (Fig. 2b). While the patient experienced grade II neutropenia from days 8–10 of each cycle, it abated spontaneously without administration of the granulocyte colony-stimulating factor. He did not develop anemia or thrombocytopenia. Moreover, owing to the preventive therapy, he did not experience nausea or loss of appetite. After four cycles, all recurrent tumors completely disappeared from the CT scan. The serum NSE level decreased to 8.0 ng/ml. The patient continued to receive maintenance atezolizumab therapy (1200 mg, administered intravenously) every 21 days, without any evidence of recurrence over the 12 month follow up. Retrospectively, programmed death ligand 1 (PD-L1) staining was performed using a cystectomy specimen. The immune cells infiltrating the tumor were positive for PD-L1, whereas the tumor cells were negative for PD-L1 (Fig. 4). The proportion of tumor-infiltrating immune cells expressing PD-L1 in the tumor area was more than 1% and less than 5%. Additionally, the proportion of tumor cells expressing PD-L1 in all tumor cells was less than 1%.

## Discussion and conclusions

SCBC is a rare tumor variant, accounting for approximately 1% of all bladder cancers; SCBC exhibits the same pathological features of SCLC and is characterized by an aggressive behavior [1, 2]. However, treatment guidelines have not yet been established due to its rarity. Its treatment algorithm was derived from the SCLC guidelines and retrospective studies [3]. Given its high malignancy, a multimodal approach was recommended as a treatment option for localized SCBC [7]. A previous retrospective study by Lynch et al. demonstrated that neoadjuvant chemotherapy before cystectomy resulted in pathological downstaging and improved prognosis compared to cystectomy without neoadjuvant chemotherapy [8]. A platinum-based regimen was recommended according to the SCLC guidelines [3, 9]. In our patient, examination of the TURBT specimen revealed muscle invasive bladder cancer, but we did not evaluate the pathological variant. Since the diagnosis of SCBC was confirmed after cystectomy, the patient did not receive neoadjuvant chemotherapy. Contrastingly, Choong et al. demonstrated that adjuvant chemotherapy after cystectomy should be considered for patients with stage III/IV disease [7]. Our patient received adjuvant chemotherapy (often used for urothelial carcinoma) due to lymph node metastasis. However, disease progression could not be prevented, even when the regimen included cisplatin

It has been well documented that SCBC has a high risk of recurrence and metastasis and is often diagnosed at an advanced stage [1, 2]. While platinum-based chemotherapy used for extensive-disease SCLC has been recommended as a treatment option for metastatic SCBC, prognosis has still been poor [3]. Ismaili et al. demonstrated that the median overall survival of metastatic SCBC is 7.6 months [3]. Horn et al. revealed that the carboplatin, etoposide, and atezolizumab regimen followed by maintenance atezolizumab monotherapy was more effective compared to the traditional regimen (carboplatin and etoposide) as a systemic therapy for extensive-disease SCLC [4]. Atezolizumab induces a blockade of PD-L1 that is expressed on the surface of tumor cells in many cancer types; PD-L1 binds to its receptor, programed death 1, and suppresses T-cell immunity against cancer [10]. Atezolizumab restores T-cell activity by inhibiting PD-L1 [10]. In our case of pure SCBC, chemotherapy combined with atezolizumab achieved a complete response. Moreover, unlike our patient, the cases of SCBC mixed with urothelial carcinoma are more frequently seen than pure SCBC in clinical practice [3, 11]. Galsky et al. demonstrated that addition of atezolizumab to platinum-based chemotherapy also improved prognosis of patients with metastatic urothelial carcinoma [12]. Chemotherapy combined with atezolizumab may also be efficacious as a treatment option for patients with SCBC of mixed pathological variants. In our case, PD-L1 expression was slightly found in the tumor-infiltrating immune cells, but not in the tumor cells. Unlike non-small cell lung cancer, small cell carcinoma has been demonstrated to exhibit PD-L1 expression more frequently in tumor-infiltrating immune cells than in tumor cells [13]. Moreover, Liu et al. revealed that patients with extensive-disease SCLC benefited from the addition of atezolizumab to chemotherapy, regardless of PD-L1 immunohistochemistry [14]. Their findings are inconsistent with our experience in this case. Comparable to our findings, Hossain et al. reported that SCBC with muscle metastasis treated with a similar therapy achieved at least four months of disease control [5]. However, only a few cases have reported similar findings. It remains unclear what biomarkers are associated with the response to atezolizumab. Furthermore, maintenance immunotherapy has attracted attention. Powles et al. revealed that maintenance monotherapy with avelumab, a PD-L1 inhibitor, following platinum-based first-line chemotherapy improved prognoses of metastatic urothelial cancer [15]. Our patient with SCBC did not derive benefits from adjuvant platinum-based chemotherapy, which is often used for urothelial cancer, but greatly responded to chemotherapy combined with atezolizumab, which is often used for extensive-disease SCLC. Based on the study protocol of extensive-disease SCLC, following atezolizumab maintenance monotherapy was administered and it achieved long-term progression free survival [4]. Further studies are required to establish the optimal maintenance therapy for SCBC.

Additionally, Horn et al. documented that the combined regimen with carboplatin, etoposide, and atezolizumab was as safe as the traditional regimen (carboplatin and etoposide) for extensive-disease SCLC because the rates of side effects were comparable in the two groups [4]. Nausea (31.3%), vomiting (12.6%), and loss of appetite (19.7%) are commonly observed adverse events of grades I/II [4], whereas neutropenia (22.7%), anemia (14.1%), and thrombocytopenia (10.1%) are commonly observed adverse events of grades III /IV [4]. Our patient only experienced grade II neutropenia. He continued to receive maintenance immunotherapy in an outpatient setting, without any serious adverse events.

In conclusion, the present report describes a case of metastatic SCBC successfully managed with carboplatin, etoposide, and atezolizumab. The regimen may be useful as a treatment option for SCBC, which is highly aggressive. However, further studies are required to evaluate the efficacy and safety of this treatment regimen at a population level and establish additional treatment strategies for SCBC.

## Data Availability

Data sharing is not applicable to this article as no datasets were generated or analyzed during the current study.

## Abbreviations

CT: Computed tomography
NSE: Neuron-specific enolase
PD-L1: Programed death ligand 1
SCBC: Small cell bladder carcinoma
SCLC: Small cell lung cancer
TURBT: Transurethral resection of bladder tumor
